# Omics approaches to understand the *MADS-box* gene family in common bean (*Phaseolus vulgaris* L.) against drought stress

**DOI:** 10.1007/s00709-024-01928-z

**Published:** 2024-01-19

**Authors:** Aybüke Okay, Tarık Kırlıoğlu, Yasin Şamil Durdu, Sanem Şafak Akdeniz, İlker Büyük, E.Sümer Aras

**Affiliations:** 1 Department of Biology, Faculty of Science, Ankara University, Ankara, 06100 Turkey; 2 Kalecik Vocational School Plant Protection Program, Ankara University, Ankara, 06100 Turkey; 3 Department of Biology, Faculty of Science, Ankara University, Block A, Emniyet, Dögol Cd. 6A, Yenimahalle, Ankara, 06560 Turkey

**Keywords:** Common bean, MADS-box, Transcription factor, Stress responses, Expression profiles

## Abstract

*MADS-box* genes are known to play important roles in diverse aspects of growth/devolopment and stress response in several plant species. However, no study has yet examined about *MADS-box* genes in *P. vulgaris*. In this study, a total of 79 *PvMADS* genes were identified and classified as type I and type II according to the phylogenetic analysis. While both type I and type II PvMADS classes were found to contain the MADS domain, the K domain was found to be present only in type II PvMADS proteins, in agreement with the literature. All chromosomes of the common bean were discovered to contain *PvMADS* genes and 17 paralogous gene pairs were identified. Only two of them were tandemly duplicated gene pairs (*PvMADS-19/PvMADS-23* and *PvMADS-20/PvMADS-24*), and the remaining 15 paralogous gene pairs were segmentally duplicated genes. These duplications were found to play an important role in the expansion of type II *PvMADS* genes. Moreover, the RNAseq and RT-qPCR analyses showed the importance of *PvMADS* genes in response to drought stress in *P. vulgaris.*

**Supplementary Information:**

The online version contains supplementary material available at 10.1007/s00709-024-01928-z.

## Introduction

In higher plants, transcription factors (TFs) have a range of functions during their life cycle. They are involved in the growth, development, morphogenesis, and stress responses of plants by binding to cis-acting regulatory sequences (Singh et al. 2002). A group of transcription factors known as *MADS-box* genes are present in eukaryotes like plants, yeasts, insects, amphibians, and mammals. The genes MONOCHROME HOME MAINTENANCE 1 (MCM1) from yeast, AGAMOUS (AG) from Arabidopsis, DEFICIENT (DEF) from Antirrhinum, and serum response factor (SRF) from humans collectively give rise to the name of MADS-box family (Riechmann and Meyerowitz 1997). *MADS-box* genes can be divided into two primary classes, type I and type II, in plants, mammals, and fungi (Ma et al. 2017). Compared to type I (M type) classes, type II (MIKC type) genes have three extra functional domains: (1) dimerization-inducing keratin-like helix (K) domain, (2) DNA-binding/dimerization domain, and (3) C-terminal domain that participates in the creation of tertiary or quaternary protein complexes during transcriptional activation contribute to functional specificity (Theißen et al. 1996; Ayra et al. 2021). Type I and type II genes are further divided into the subclasses based on structural variations (Henschel et al. 2002).

*MADS-box* genes play a vital role in diverse essential developmental processes, including flower structure and root development, fruit growth, timing of flowering, and regulation of gametophyte cellular division (Liljegren et al. 2000; Michaels et al. 2003; Zhang et al. 2008; Martel et al. 2011). The majority of the proteins related to flower organ development are regulated by *MADS-box* genes in plants (Coen and Meyerowitz 1991; Pelaz et al. 2000; Thei and Saedler 2001; Pinyopich et al. 2003; Ditta et al. 2004).

In previous studies, it was shown that *MADS-box* genes had the ability to respond to diverse abiotic stress conditions (Wei et al. 2014). In a study by Zhang et al. (2012), *MADS-box* genes were found to be differentially expressed in response to ABA, cold, salt, and drought stresses in maize (Zhang et al. 2012). Another study in rice showed that *MADS-box* genes were associated with pathogen resistance and drought tolerance (Lee et al. 2008b; Khong et al. 2015). In the study of Chen et al. (2019), transgenic *CaMADS-*expressing pepper plants have been found to be more resilient to cold, high salt, and mannitol stresses than wild type (Chen et al. 2019). Additionally, *MADS-box* genes linked to stress tolerance have been found in other species, such as wheat, sheep grass, chinese cabbage, and tomato (Saha et al. 2015; Guo et al. 2016; Jia et al. 2018; Schilling et al. 2020). The findings related to the function of *MADS-box* genes for biotic and abiotic stress tolerance offer valuable resources for their possible utilization in plant breeding and enhancing crop production. Nonetheless, there is limited information regarding the *MADS-box* genes in common bean in the literature. However, common bean is the most widely cultivated and produced edible legume globally which is consumed as a fresh vegetable or dried grain (Anlarsal et al. 2000; Idiku et al. 2022). Bioinformatics analysis was used to identify and characterize the *MADS-box* gene family members in common bean in this study. In addition, the role of *PvMADS* genes in response to drought stress was evaluated at mRNA level for the first time using RNAseq and RT-qPCR analyses.

## Materials and methods

### Identification of MADS-box proteins in *P. vulgaris*

MADS-box sequences of *P. vulgaris* were acquired from the Phytozome (https://phytozome-next.jgi.doe.gov/) through Pfam (PF00319) code search and the sequences were deposited in Supplementary Table 1 (Goodstein et al. 2012). For characterization of putative proteins, candidate *P. vulgaris* MADS-box proteins were used as query sequences in NCBI blastp (https://blast.ncbi.nlm.nih.gov/Blast.cgi). Redundant sequences were removed using the decrease redundancy tool (http://web.expasy.org/decrease_ redundancy/), and sequences were checked for MADS box domains using HMMER (http://www.ebi.ac.uk). Physical and chemical properties were calculated using Expasy ParatProm (www.web.expasy.org/protparam). Domains of MADS box proteins were discovered using InterProScan (https://www.ebi.ac.uk/interpro/search/sequence/). The WoLF PSORT: Protein Subcellular Localization Estimator Tool (https://wolfpsort.hgc.jp/) was used to determine where PvMADS proteins were located. Wolfpsort is a tool used to predict the cell nucleus localization of a protein. Based on databases and calculations, Wolfpsort estimates the probability at which site within the cell a protein is located. The ratios obtained as a result of Wolfpsort analysis usually express the probabilities for specific sub-cellular regions of the protein.

### Phylogenetic analysis

The peptide sequences of Arabidopsis and common bean MADS-box proteins were aligned in MEGA11 using ClustalW algorithm (Hall 2013; Thompson et al. 2003). The phylogenetic tree was then created using the maximum-likelihood (ML) approach with 1000 bootstrap value using MEGA11 software (Tamura et al. 2021). The phylogenetic tree was ultimately visualized using iTOL (https://itol.embl.de/) online tool (Letunic and Bork 2007).

### Chromosomal distribution and duplication analysis of *PvMADS* genes

The *P. vulgaris* genomic feature file (GFF3) was downloaded from the JGI Data Portal, and chromosomal locations were plotted using TBtools software (http://cj-chen.github.io/tbtools/). The genomes of specific organisms were acquired from Phytozome v13, and orthologous gene pairs were identified using TBtools MScanX module. In order to comprehend the syntenic connections between *MADS-box* genes, Dual Synteny Plotter software was utilized to create synteny maps. Collinearity analysis confirmed paralog relationships and then visualized with the Circos tool in TBtools software (Chen et al. 2020). The CLUSTALW program was used to predict the peptide sequences of the transcribed *PvMADS* genes. The Ka/Ks calculator was utilized to evaluate the selection pressure. Therefore, the veracity of the identified duplication connections was verified utilizing this information (Suyama et al. 2006).

### Gene structure, protein motifs, and homology analysis

The exon–intron pattern of *PvMADS* genes was visualized using the GFF3 file in Tbtools software (Chen et al. 2020). MEME (https://meme-suite.org/meme/) was used for motif extraction of PvMADS proteins (Bailey et al. 2006). Sequence of Motif 1, three-dimensional structure, was obtained using AlfaFold (Jumper et al. 2021).

### miRNA and promoter prediction of *PvMADS* genes

To investigate the cis-regulatory components in the promoter region, the 1.5 kb genomic sequence of each *PvMADS* gene was extracted from the Phytozome v13 database. Potential cis-regulatory elements in the promoter sequence were evaluated using the PlantCARE online tool (https://bioinformatics.psb.ugent.be/webtools/plantcare/html/). Potential miRNA prediction was obtained using the psRNATarget (http://plantgrn.noble.org/psRNATarget) online tool. *PvMADS* genes targeted by miRNAs were then visualized using Cytoscape 3.9.1 program (Smoot et al. 2011).

### Investigation of expression level of *PvMADS* genes in several tissues and under drought

Expression levels of *PvMADS* genes in several plant organs at different growth stages were retrieved from Phytozome v13. The expression levels in silico were measured using FPKM (fragments per kilobase million). The FPKM values were transformed into log_2_FC values and a heatmap was then generated utilizing the Heatmap module in TBtools. Expression levels of *PvMADS* genes under drought stresses were calculated using RNA-seq data from the sequence reading archive (SRA) by Illumina sequencing. SRA data with accession numbers SRR8284481 (drought stress leaf) and SRR8284480 (control leaf) were used as previously described by Aygören et al. (2023). Hierarchical clustering heatmaps were created using TBtools.

### Plant materials, environmental factors, and stress management

In this study, “Zülbiye,” a Turkish common bean variety, was included in experimental studies since it has been shown to be sensitive to various abiotic stress factors such as drought and high salinity in previous studies (Yıldız et al 2021; Güler et al. 2012). The plant seeds were gifted by “Traditional Regional Agricultural Research Institute in Eskişehir, Turkey.” Followingly, uniform looking seeds were selected and kept in distilled water for 2 h prior to sowing into the weight adjusted pots. Then, the pots were taken into controlled plant growing chamber which was set to + 25 °C, 70% humidity, and 20000 LUX light exposure (16 h day, 8 h night). From the first day, the shelf positions of the pots in the growing chamber were changed regularly. On the 8th day after planting, plants were separated into two groups, and control group plants were continued to be watered; however, water was withheld for stress group plants. Plant weights were taken regularly every 2 days from the first day of stress application. At the end of the 16th day, as the control group plant samples were continued to be watered, an increase was observed in the growth, height, and weight of the leaves. The stress-exposed plants were observed to be shorter than the control group plants; their leaves were curved and pale, and their weights continuously dropped because of water loss. Followingly, the plants were sampled and were crushed with liquid nitrogen in a mortar and then stored at − 80 °C (Büyük and Aras 2017; Aygören et al. 2023).

### RNA isolation, cDNA synthesis, and qPCR analyzes

RNA was extracted using the Machery-Nagel NucleoSpin® RNA Kit. RNAs were then subjected to quality control analysis using NanoDrop Lite Spectrophotometer (NanoDrop Technologies, Wilmington, DE, USA) and agarose gel electrophoresis. For complementary DNA synthesis, the Roche (USA) cDNA synthesis kit was employed. PrimerQuest Tool (https://www.idtdna.com) was used to construct the primers, and primer sequences were presented in Supplementary Table 6. iTaq Universal SYBR Green Supermix (Biorad, USA) was used for RT-qPCR reactions following the conditions previously suggested by Büyük et al. (2022). The Light Cycler® Nano qPCR Instrument was used to perform RT-qPCR reactions (Roche, USA). The ACT (Actin) gene was employed for the standardization of RT-qPCR data through the 2^−∆∆CT^ formula (Livak and Schmittgen 2001). GraphPad Prism 7 software was used for statistical analysis, and Fisher’s least significant difference test at 0.05 significance level was applied with two-way ANOVA method.

## Results and discussion

### Identification, characterization, and phylogenetic analysis of MADS-box genes in *P. vulgaris*

Common bean (*P. vulgaris* L.) is a seasonal vegetable that is high in protein, vitamins, fiber, and antioxidants. For hundreds of years, it has been grown for human food with its green or dried parts all over the world (Vougeleka et al. 2023). Recently, the developing high-quality varieties through novel breeding technologies, enhancing production and quality efficiency, have become important research areas in agricultural biotechnology (Naqvi et al. 2022).

Sequencing technologies have enabled the rapid release of genome sequences, and as such, many gene families have become the focus of attention in recent years. Transcription factor gene families have gained popularity in agricultural biotechnology for the last decades since they interact with cis-acting regions of the genes to control the expression of gene of interest. The structure of *MADS-box* genes in several plant species has been well studied, and previous studies have indicated the importance of MADS-box transcription factors in growth and abiotic stress response of plants (Parenicova et al. 2003; Zhao et al. 2011). Due to many of these reasons, the *MADS-box* gene family has the potential to be a valuable resource in promoting the development of genetically modified crops and conventional breeding techniques to improve agricultural yield (Lee et al. 2008b; Khong et al. 2015; Theisse and Becker 2003).

The *MADS-box* gene family has been systematically characterized in diverse plants up to date, including *Arabidopsis thaliana* (*n* = 109) (Parenicova et al. 2003), *Populus trichocarpa* (*n* = 105) (Leseberg et al. 2006), *Solanum tuberosum* (*n* = 156), *Solanum lycopersicum* (*n* = 131) (Zou et al. 2017), *Glycine max* (*n* = 106), *Malus domestica* (*n* = 146) (Tian et al. 2015), *Oryza sativa* (*n* = 75) (Arora et al. 2007), *Vitis vinifera* (*n* = 32) (Díaz-Riquelme et al. 2009), *Cucumis sativus* (*n* = 43) (Hu and Liu 2012), *Prunus mume* (*n* = 80) (Xu et al. 2014), *Zea mays L.* (*n* = 75) (Zhang et al. 2012), and *Sorghum bicolor* (*n* = 65) (Zhao et al. 2011).

In this study, 79 *MADS-box* genes have been identified in common bean genome, and they were named from *PvMADS-01* to *PvMADS-79* according to their chromosomal positions (Supplementary Table 1). Moreover, physico-chemical properties of PvMADS proteins were determined using their peptide sequences. Accordingly, the length and isoelectric points (pI) of PvMADS proteins were found to be ranged from 88 (*PvMADS-41*) to 381 (*PvMADS-03*) and from 4.6 (*PvMADS-03*) to 9.8 (*PvMADS-41*), respectively. In comparison, 58 PvMADS proteins were determined to be basic (pI > 7) and 21 PvMADS proteins to be acidic (pI < 7) with molecular weights ranging from 10.15 (*PvMADS-41*) to 42.02 (*PvMADS-03*) kDa.

In this study, three protein characteristics were also evaluated: (1) aliphatic index, (2) instability index, and (3) hydropathicity (GRAVY). The aliphatic index (1) measures the number and proportion of aliphatic (hydrocarbon-containing) amino acids in a protein’s amino acid sequence. This is used to understand the structural properties and functions of proteins. Aliphatic amino acids can form the hydrophobic internal regions of proteins or be associated with the membrane permeability of proteins (Avrahami et al. 2001). In the current study, the aliphatic index values of PvMADS proteins were found to be between 61.58 (PvMADS-10) and 98.59 (PvMADS-15).

The instability index (2) measures the instability of a protein, and it is used to determine which regions of a protein can change or transform rapidly in a test tube. This is important for identifying the variable regions of proteins and understanding their function. The proteins with instability index values less than 40 are referred to as stable proteins; however, they are known as unstable proteins with an instability index value more than 40 (Guruprasad et al. 1990). In this study, the instability index values of PvMADS proteins were found to vary between 30.12 (PvMADS-67) and 73.88 (PvMADS-17).

The GRAVY score (3) measures the hydrophobicity or hydrophilicity of a protein. Positive GRAVY scores can indicate a hydrophobic protein region, while negative GRAVY scores can indicate a hydrophilic protein region. Moreover, the GRAVY score is used to understand the solubility of proteins and their cellular localization. In this study, the GRAVY scores except for PvMADS-11 protein were found to be negative which means that PvMADS proteins were hydrophilic.

In addition, the subcellular localization prediction of MADS-box proteins, which is helpful for understanding protein function, was also performed, and most of the PvMADS proteins were found to be localized in the nucleus in this study (Supplementary Table 1). These ratios give clues about cellular localization by indicating in which cell sub-region a protein is more likely to be found. Similar to our findings here, MADS-box proteins were also reported to be located in nucleus in *Argania spinosa* by Louati et al. (2021) and in *Setaria italica* L. by Zhao et al. (2021) in recent years.

**Fig. 1:**
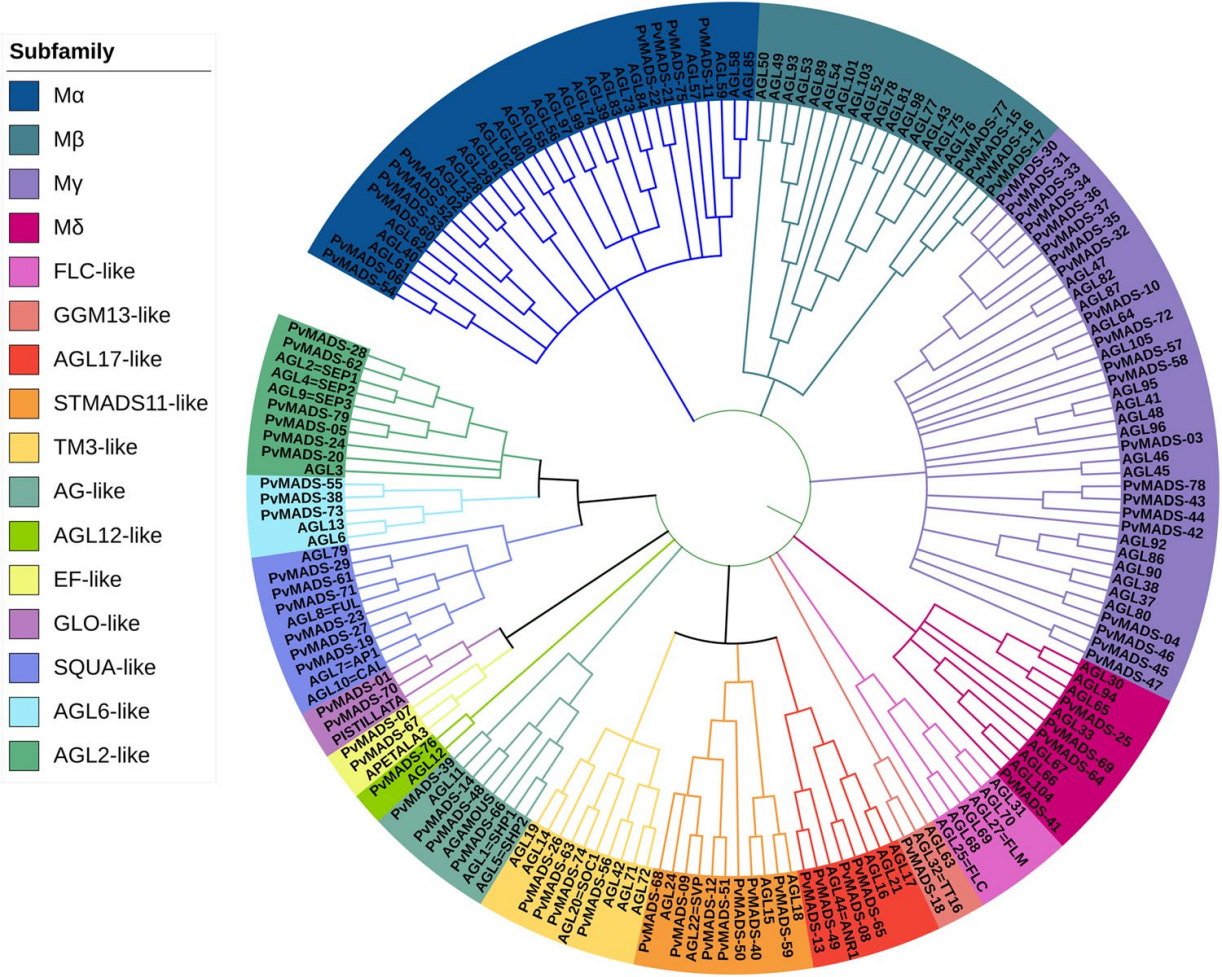
*P. vulgaris* and *A. thaliana* MADS-box protein family phylogenetic analysis. The evolutionary tree produced by means of the ML (maximum likelihood) estimation method in MEGA11 and displayed on iTOL

To investigate the relationships between MADS-box proteins, a phylogenetic tree was constructed using Arabidopsis and common bean MADS-box peptide sequences (Fig. 1). According to the phylogenetic tree, PvMADS proteins were found to be classified into two main classes as type I (M type (10 Ma, 4 Mβ, 21 Mγ, 4 Mδ)) and type II (MIKC type (2 GLO-like, 6 AGL2-like, 2 EF-like, 4 AGL17-like, 7 STMADS11-llike, 4 AG-like, 1 GGM13-like, 6 SQUA-like, 4 TM3-like, 3 AGL6-like, 1 AGL12-like) in accordance with the previous findings of Parenicova et al. (2003) in Arabidopsis. However, no FLC-like genes, which are known to be control the flowering time, have been identified in *P. vulgaris* in this study. In most recent studies, Cheng et al. (2023) and Zhang et al. (2023) have also been revealed the absence of FLC-like genes in *S. edule*, *Z. mays*, *S. bicolor*, *S. italica*, rice, *B. distachyon*, and *Z. latifolia* plant species.

Recent studies have indicated that the number of type I *MADS-box* genes decreased dramatically during evolution of monocotyledons (Arora et al. 2007; Zhao et al. 2021). However, in this study, the number of type I (*n* = 39) and type II (*n* = 40) *MADS-box* genes were very close to each other as contradictory to this statement. In the literature, some studies on rice and Arabidopsis showed that type I genes had a faster birth-and-death evolution rates than type II genes. Gene duplication was suggested to be the main reason of the greater birth rate of type I *MADS-box* genes, and despite type I genes’ high rate of extinction during evolution, their quantity has been maintained due to duplications (Nam et al. 2004).

### *PvMADS* gene chromosomal locations and gene duplication analyses

**Fig. 2:**
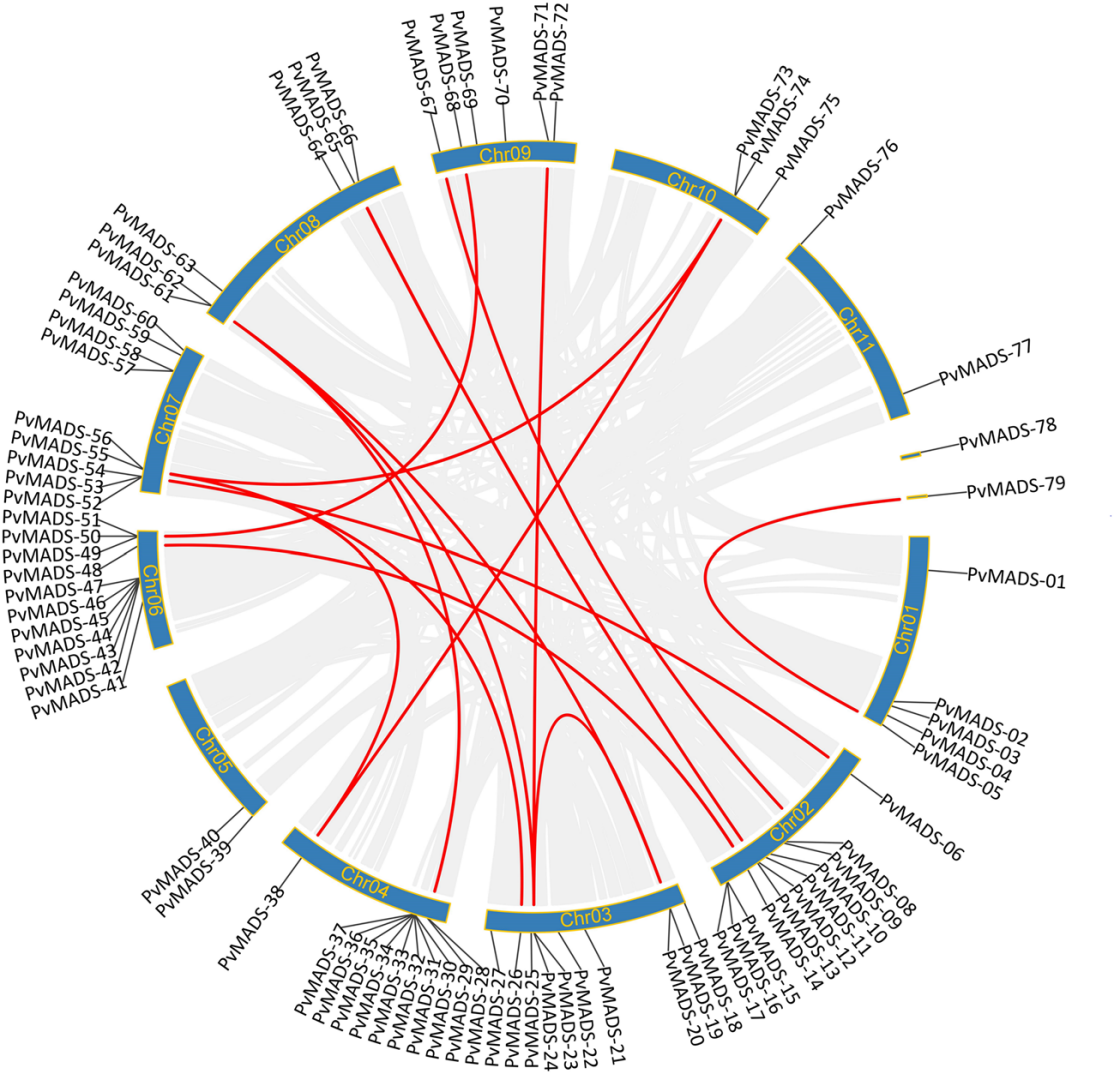
Circos display and chromosomal localization of *PvMADS* genes. Gray lines display all of the synteny blocks in the *P. vulgaris* genome, whereas red lines depict the duplicated *PvMADS* gene pairs

Physical locations of *MADS-box* genes on *P. vulgaris* chromosomes were visualized with TBtools software. *PvMADS* genes showed an unequal distribution across all chromosomes of common bean (Fig. 2). Chromosome 2 was the chromosome with highest number of *PvMADS* genes (*n* = 12). The distribution of the remaining *PvMADS* genes onto chromosomes was as following: 11 *PvMADS* genes on chromosome 4 and 6; 10 *PvMADS* genes on chromosome 3; 9 *PvMADS* genes on chromosome 7; 6 *PvMADS* genes on chromosome 8; 2 *PvMADS* genes on chromosome 11; 3 *PvMADS* genes on chromosome 10; and 2 *PvMADS* genes on chromosome 5. Meanwhile, two *PvMADS* genes were found to be present on scaffolds. According to Deng et al. (2023), the exact chromosomal locations of the five *PfMADS* genes (*Paulownia fortunei*) could not be ascertained as they were present on scaffolds that had not been stabilized yet (Deng et al. 2023). Similar to the distribution pattern of *PvMADS* genes in this study, Lakhwani et al. (2022) also reported that the *MADS-box* genes distributed at different rates across all chromosomes in *Musa balbisiana* (Lakhwani et al. 2022). According to some studies in eukaryotes, if genes are more widely spread across the genome rather than densely packed on a small number of chromosomes, the organism’s complexity will rise (Toor and MD 2019). Besides, distribution of a gene in different proportions on chromosomes can affect the biological functions, adaptability, and phenotype of the organism. As can be understood, the distribution of gene family members on different chromosomes is important for these gene members to gain different functions (Wang 2020).

Gene duplication are regarded as a crucial element in the evolution of eukaryotes (Taylor and Raes 2004). The discovery of new genes, the development of novel functions, and the extension of gene families are all made possible by the important mechanism of gene duplication. Adaptation of plants to environmental factors, development of new organs, or interactions at the molecular level can appear as evolutionary novelties (Magadum et al. 2013). For example, intensive duplication events have led to expansion of *MADS-box* genes. Many *MADS-box* genes have undergone duplication events and differ in their functions (Panchy et al. 2016).

In this study, it was aimed to uncover the molecular mechanism behind the expansion of the *PvMADS* gene family by detecting paralog genes. Accordingly, 17 paralogous gene pairs were identified, and only two of them were tandemly duplicated gene pairs (*PvMADS-19/PvMADS-23* and *PvMADS-20/PvMADS-24*) (Fig. 2, Supplementary Table 2). The remaining 15 paralogous gene pairs were segmentally duplicated genes (Fig. 2). Ninety-four percent of the detected duplications was observed between type II *PvMADS* genes. Tandem duplications have been only detected in type II (MIKC) class *PvMADS* genes, and these were the genes from SQUA-like (*PvMADS-19* and *PvMADS-23*) and from AGL2-like subfamilies (*PvMADS-20* and *PvMADS-24*). This result might be related to late emergence and differentiation of type I genes compared to type II genes during evolutionary time (Zhou et al. 2023). Unsurprisingly, segmentally duplicated gene pairs were classified under same subfamilies in the phylogenetic tree such as *PvMADS-05* and *PvMADS-79* from AGL2-like, *PvMADS-13* and *PvMADS-65* from AGL17-like, and *PvMADS-50* and *PvMADS-68* from STMADS11-like subfamilies. The Ka/Ks ratio can be considered as an indicator of selection pressure during the evolution. Therefore, the Ka/Ks ratio of all homologous genes was calculated to discover the selection pressure on the evolution of *MADS-box* genes in common bean (Supplementary Table 2). The Ka/Ks ratio was below “1” for all duplications which indicated that the *MADS-box* genes in the common bean were under strong purifying selection. In a previous study on *Camellia sinensis*, all of the duplicated MADS-box gene pairs’ Ka/Ks values were also found to be less than 1 similar to our findings here (Hu et al. 2023).

**Fig. 3:**
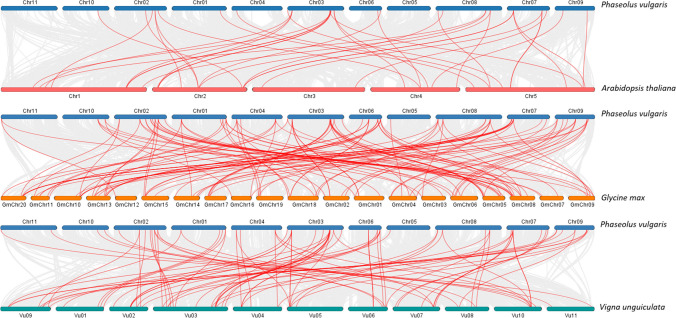
Synteny analysis of associated genes between *P. vulgaris* and *A. thaliana*, *G. max*, and *V. unguiculata.* The background displays gray lines indicating the presence of identical linear blocks in the genome of common bean and other plants. The paired syntenic *MADS-box* genes are highlighted with red lines

Orthologs are genes that have developed in various species from a common ancestral gene through speciation and typically retain the same function over time. Identification of orthologs is a critical process for better understanding the gene function (Koonin 2005). In this study, orthologous relationships of *MADS-box* genes between *P. vulgaris* and other genomes (*Arabidopsis thaliana*,* Glycine max*, and *Vigna unguiculata*) were examined (Fig. 3). As a consequence, there were 38, 61, and 52 orthologous gene pairs between *P. vulgaris* and those other plants, Arabidopsis, soybean, and kidney bean (Supplementary Table 3). The high number of orthologous relationships between studied genomes might show that the *MADS-box* gene family spread mostly as a result of duplication events in plant kingdom (Airoldi and Davies 2012).

### Gene structure and conserved protein motifs of PvMADSs

**Fig. 4:**
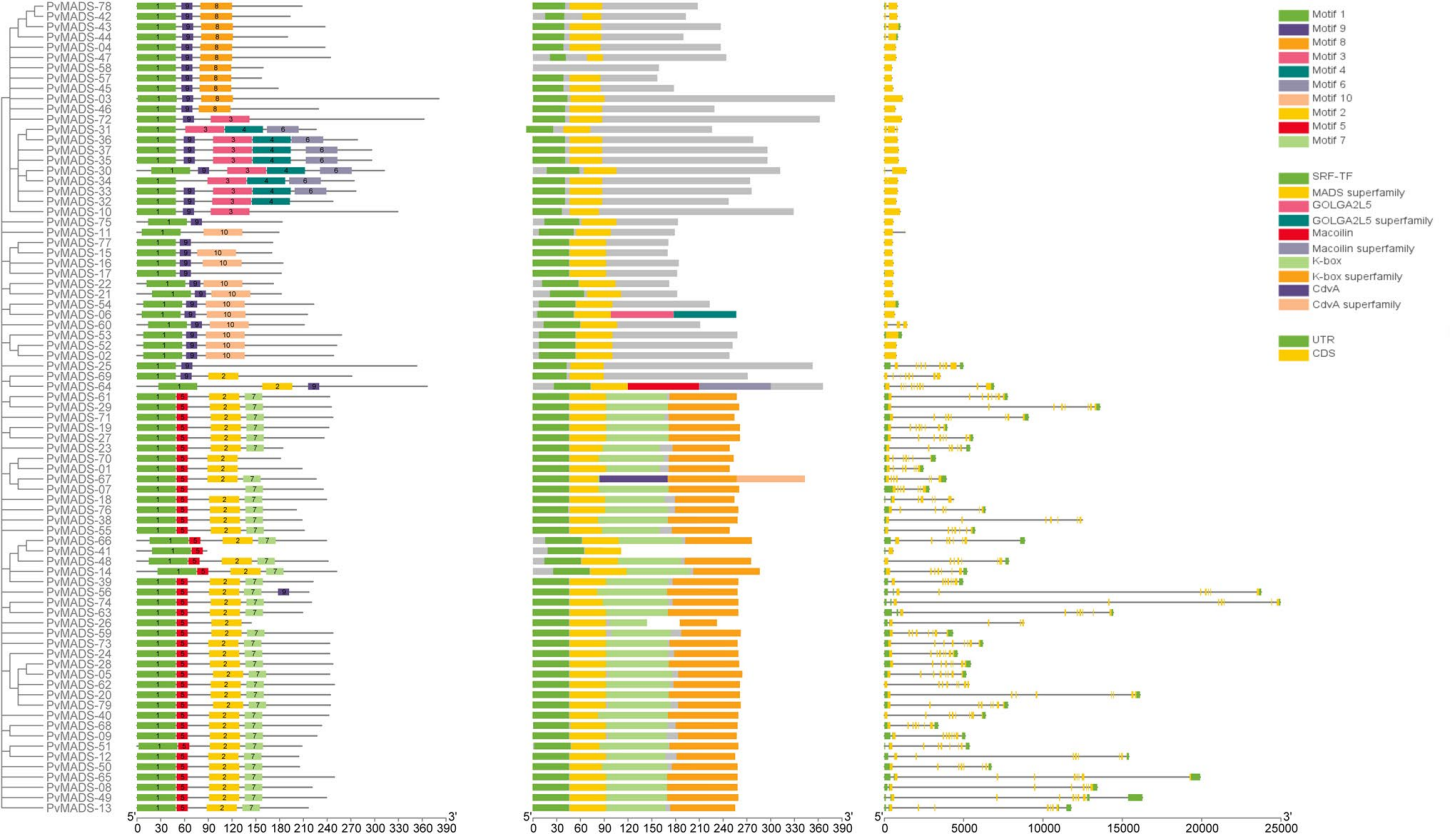
Phylogenetic relationships of PvMADS proteins based on motif, domain, and exon–intron structure

To identify conserved domains in protein structure, the peptide sequences of all PvMADS proteins were retrieved from Phytozome v13. Then, conserved domains were searched in the NCBI CDD database and visualized using TBtools (Fig. 4). The names of detected conserved domains in PvMADS proteins were SRF-TF, MADS superfamily, GOLGA2L5, GOLGA2L5 superfamily, Macoilin, Macoilin superfamily, K domain, K domain super family, CdvA, and CdvA superfamily (Fig. 4).

Human serum response factor (SRF), essential for cell proliferation and differentiation, is a ubiquitous nuclear protein. The SRF core domain plays role for DNA binding and dimerization and belongs to the MADS domain protein family (Pellegrini et al. 1995). In this study, SRF-TF domain was only found in type I *PvMADS* genes in *P. vulgaris*. This result was also compatible with results of Alvarez-Buylla et al. (2000) and Parenicova et al. (2003) who previously showed the presence of SRF-like domain proteins in type I MADS-box proteins. The MADS-box domain is commonly known to be associated with the K-box area (Norman et al. 1988). Moreover, Lu et al. (2018) reported that SRF-TF and K domain regions were conserved together between type II MADS-box genes (Lu et al. 2018). Similarly, the findings of our study showed that SRF-TF and K domain regions coexist in type II MADS-box genes.

CdvA domain is known to organize DNA-containing double-helix filaments and is thought to be involved in cell division. Moreover, CdvA, known to be in archaea, is associated with ESCRT (Moriscot et al. 2011). The ESCRT system plays a crucial role in fostering the growth and maturation of plants. In different studies on Arabidopsis, ESCRT mutants resulted in plant death (Haas et al. 2007; Gao et al. 2014; Nagel et al. 2017). GOLGA2L5, representing the conserved domain found in Golgin subfamily A members, is an unstable region enriched with segmental copies containing the core duplicon. Nuclei represent ancestral copies around which additional replication blocks have been formed and correspond to expansions of gene families, some of which show signatures of positive selection. In this study, *PvMADS-06* gene was found to contribute to the expansion of *PvMADS* gene family by establishing both paralogous and orthologous interactions and was also found to have GOLGA2L5 and GOLGA2L5 superfamily domains.

Introns have been shown to influence gene expression at several levels in higher organisms. The primary aim of introns is to translate diverse proteins by generating various combinations of exons through alternative splicing mechanism, which raises the proteome’s quality. Considering that the splicing efficiency of several tRNAs and mRNAs is differentially altered in coffee and rice plants under abiotic stress conditions (e.g., drought, cold, and heat), it was determined that introns respond to abiotic stresses by differentially regulating gene expression (Dinh et al. 2016).

The exon and intron structures of *PvMADS* genes in *P. vulgaris* genome were obtained using the General feature file (GFF3). Accordingly, the number of introns in *PvMADS* genes was found to be varied between 0 and 11 (Fig. 4). The majority of type I *MADS-box* genes were found to have only one exon with a short sequence in Arabidopsis, soybean, rice, and apple (Ren et al. 2022; Shah et al. 2022; Zhang et al. 2022b). In this study, type I *PvMADS* genes were also found to include single exon; however, type II *PvMADS* genes were found to contain more than one intron. On the other hand, different number of introns might be correlated to different evolution rates of *PvMADS* genes. Similarly, Lai et al. (2022) determined that *SiMADS* genes exhibited different exon–intron structures in *Setaria italica* recently.

**Fig. 5:**
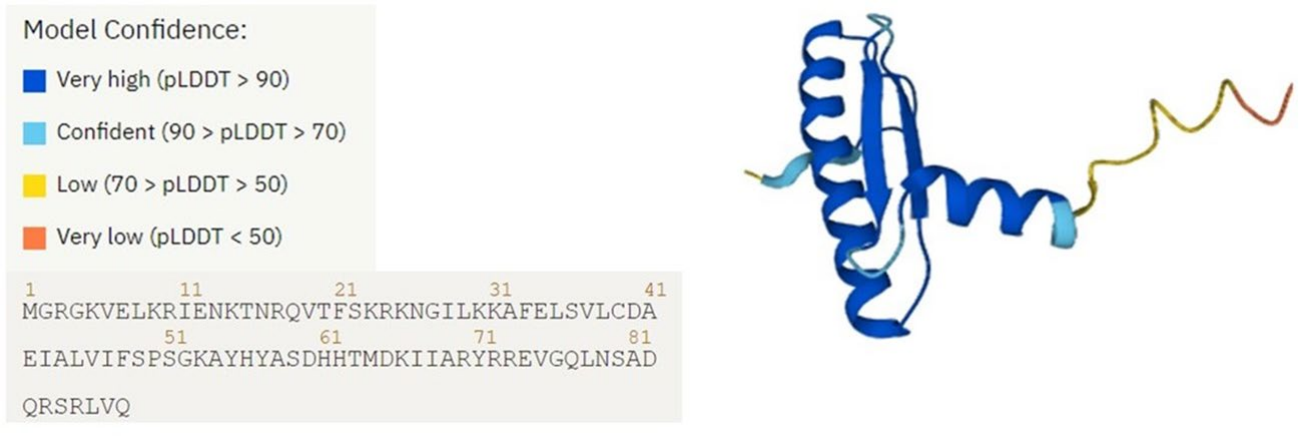
Motif 1 content, 3D structure, and expected position confidence of AlphaFold

Conserved motif structures of PvMADS proteins were also investigated in the current study. Motif 1, which was found to be conserved in all proteins, was searched using AlphaFold in order to check for the presence of MADS-box domain (Fig. 5). As a result of this analysis, the Motif 1 (MADS-box domain) was found to be composed of α-Helices and β-sheets layers and had an antiparallel beta-sheet structure. In a study of Tian et al. (2015), the conserved α-helices and β-sheet structures were also detected in apple MADS-box proteins similar to our findings. Motif composition and distribution pattern showed that PvMADS proteins from the same subfamily mostly contained similar motifs in their protein structure. Motif 8/3/4/6/10/9 were frequently seen in type I group PvMADS proteins, while motif 5/7/2 were mostly observed in type II group PvMADS proteins. Some PvMADS proteins were found to differ from other members of the same subclass in terms of certain motifs. For example, some of the Mγ subfamily *PvMADS* genes contain motif 9, while others contain motif 3/4/6. The evolution of *MADS-box* genes in *P. vulgaris* may account for these differences.

### Cis-regulatory element analysis in *PvMADS* promoters

**Fig. 6:**
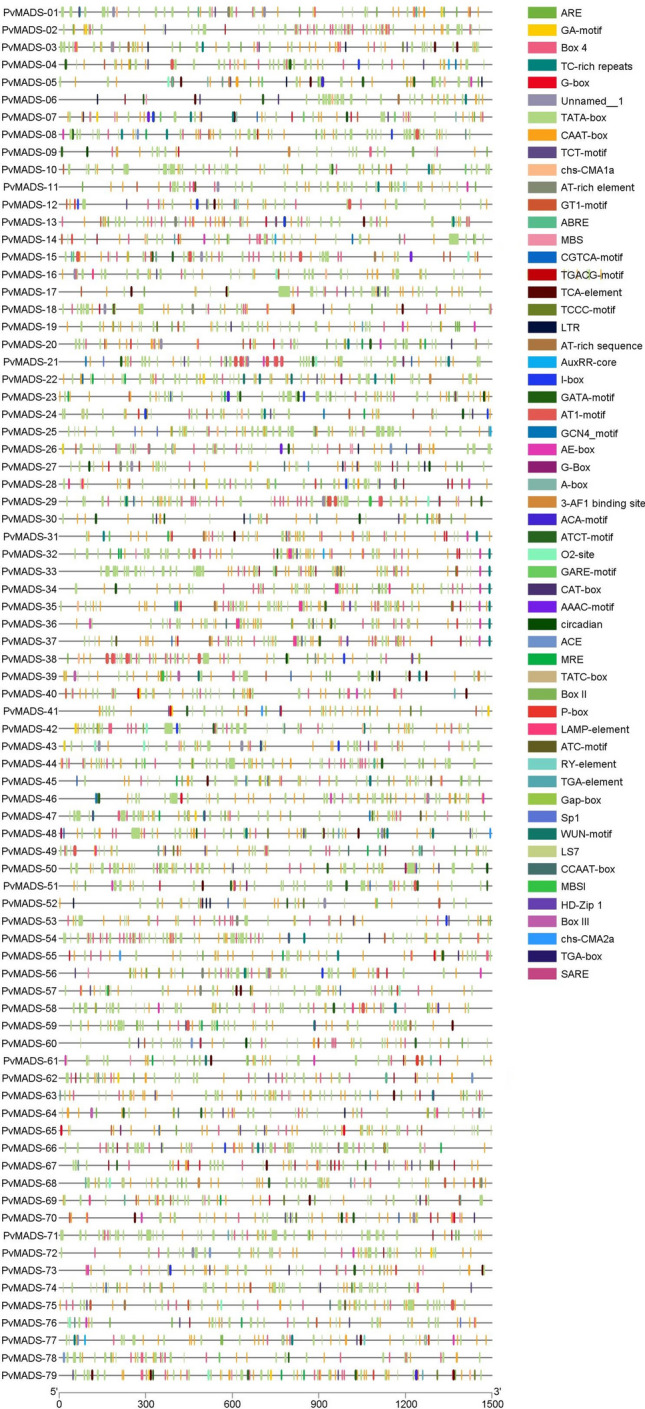
Locations of cis-regulatory elements detected in *PvMADS* genes

**Fig. 7:**
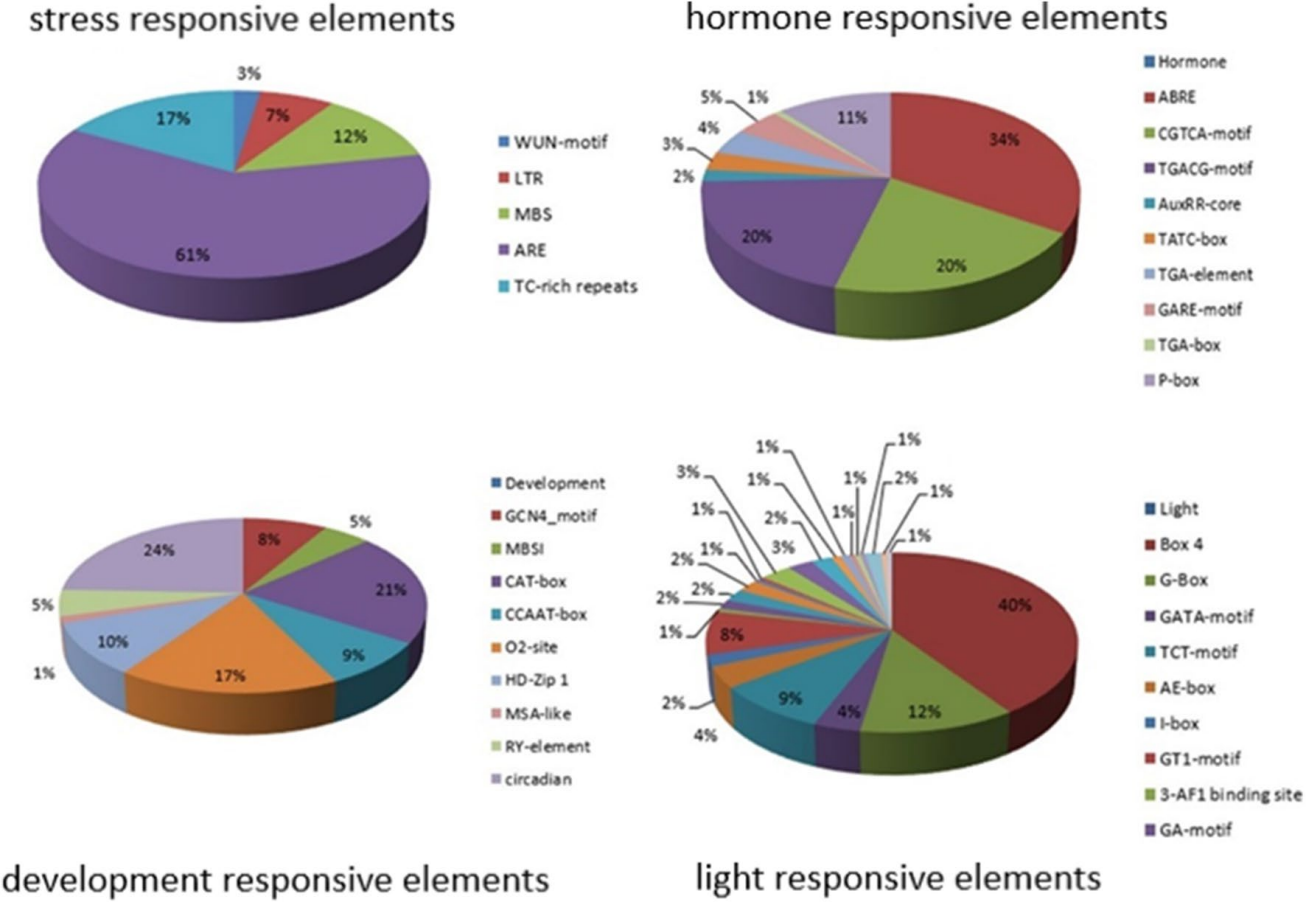
Categorized distributions of cis-regulatory elements of *PvMADS* genes

The cis regulatory elements of the *PvMADS* genes were investigated, and the following elements were found in different ratios: 88% BOX4, 73% ARE, 55% ABRE, 49% TCT motif, 43% TC-rich repeats, 32% AE-box, 29% TCA-element, 26% MRE, 25% MBS, 20% circadian, 7.5% 3-AF1 binding site, 7.5% ACE (Fig. 6, Supplementary Table 4). These cis-regulatory elements were categorized according to their functions in the cell (Fig. 7).

The WUN motif, LTR, MBS, ARE, and TC rich repeats were cis-regulatory elements belonging to the stress category. Zhao et al. (2020) found that pomegranate *MADS-box* genes also contained rich repeats of stress-responsive cis elements. Similarly, Tanin et al. (2022) showed that *MADS-box* genes, which were responsible for multi-abiotic stress tolerance in wheat, were found to contain same cis-regulatory elements in their promoter regions. Additionally, Wang et al. (2018) reported that similar cis-regulatory elements were also detected in *bZIP* genes which respond to salt and drought stress in *V. radiata* and *V. angularis*.

ABRE, CGTCA motif, GACG motif, AuxRR core, TATC box, TGA element, GARE motif, TGA box, and P box were detected cis-regulatory elements in *PvMADS* genes, and they are known to associate with hormone response in plants. ABRE participates in ABA signaling pathway and is involved in the control of diverse abiotic stressors. According to Fujita et al. (2005), ABRE was linked with drought tolerance in *Arabidopsis thaliana* (Fujita et al. 2005). Kim et al. (2011) demonstrated that the *DREB2A* gene, which regulates drought-inducible genes in Arabidopsis, contains the ABRE promoter sequence.

The detected cis regulatory elements of *MADS-box* genes in the development category were GCN4_motif, MBSI, CAT-box, CCAAT-box, O2-site, HD-Zip 1, MSA-like, RY-element, and circadian. On the other hand, the cis-regulatory elements in the light response category were Box 4, G-Box, GATA-motif, TCT-motif, AE-box, I-box, GT1-motif, 3-AF1 binding site, GA-motif, TCCC-motif, chs-CMA1a, chs-CMA2a, LS7, MRE, AT1-motif ATCT-motif, Gap-box, ACE, Sp1, Box II, AAAC-motif, LAMP-element, ATC-motif, and ACA-motif. Shariatipour and Heidari (2018) explored the genes and their governing mechanisms during the period of drought stress in Arabidopsis and found that 2558 gene entries in root and 3691 gene entries in shoot tissues exhibited considerably distinct expressions as compared to the control state. Certain cis-regulatory elements (ABRE, P-box, TATC-box, CGTCA-motif, GARE-motif, GATA- motifi, TCT-motif, GT1-motif, Box 4, G-Box, I-box, LAMP-elemanı, Sp1, MBS, TC-rich repeats, TCA-element) have been identified as the most important elements in the transcriptional regulatory activity of differentially expressed genes.

### In silico identification of miRNAs targeting *PvMADS* genes

MicroRNAs (miRNAs) are non-coding endogenous small RNAs (20–24 nucleotides long) that suppress the expression of some genes in eukaryotes at post-transcriptional level (Saini et al. 2008; Ha and Kim 2014). Furthermore, miRNAs participate in a wide range of biological mechanisms that affect plant development and biotic/abiotic response (Šečić et al. 2021).

**Fig. 8:**
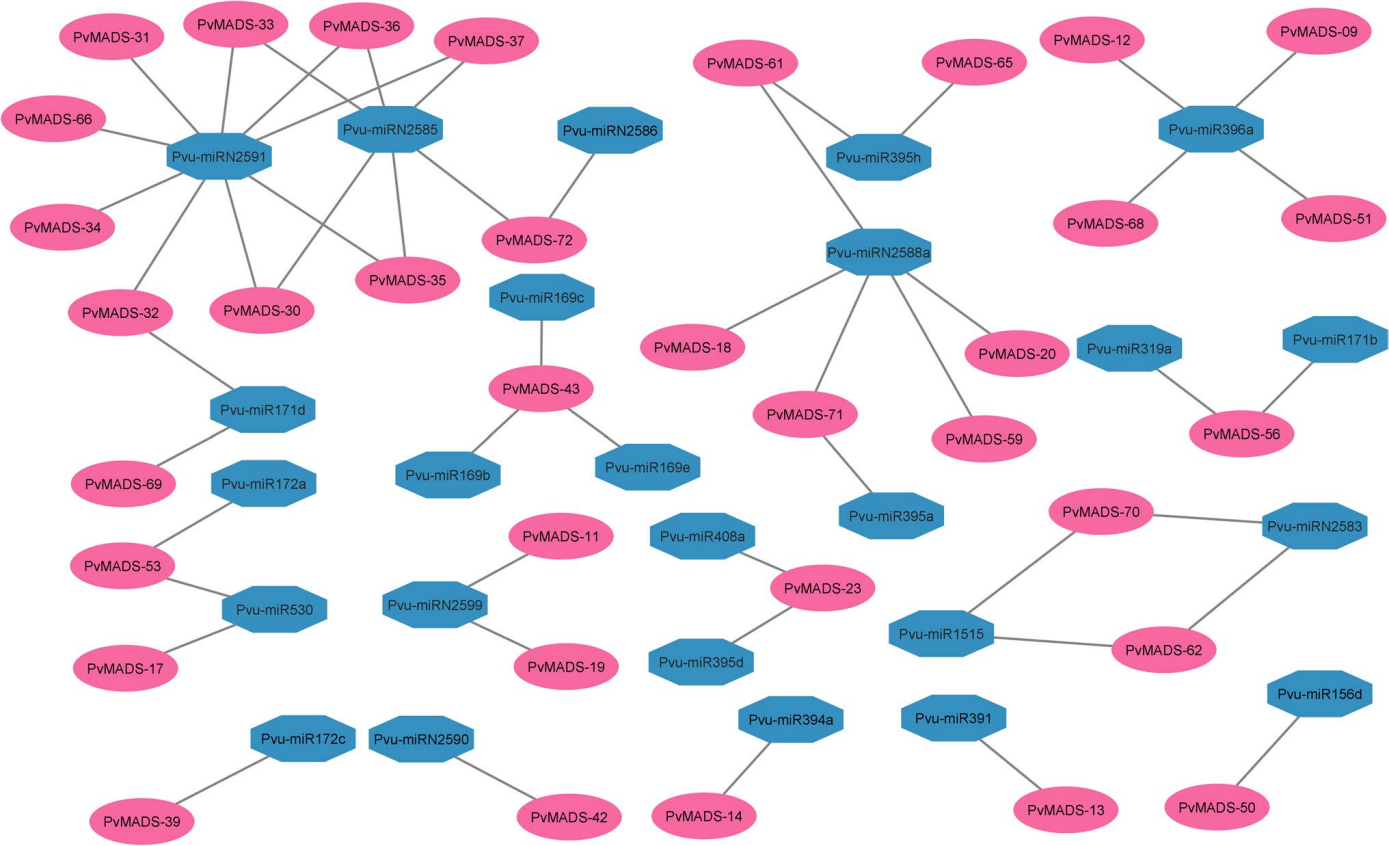
Network display of miRNAs targeting *MADS-box* genes in *P. vulgaris*

A latest research has indicated that the mechanism of miRNA-mediated regulation can enhance the agronomic characteristics of plants and confer resistance to abiotic stress, thereby promoting sustainable agricultural production. Plants experiencing stress can improve their ability to withstand against harsh environmental conditions like drought by controlling the gene expression of specific miRNAs to ensure survival and adaptation (Sun et al. 2021). Studies on miRNAs provide important information about plant stress resistance and may reveal new insights into the adaptation mechanism in plants (Zhang et al. 2022a). As a result of miRNA analysis, miRNAs targeting *PvMADS* genes were determined, and various miRNA families have been found (Fig. 8, Supplementary Table 5).

The genes targeted by miRNA-171 were involved in the development of roots and leaves, signaling via photochromes, polarity of lateral organs, formation of meristems, development of vasculature, and response to stress in *Arabidopsis thaliana* (Lee et al. 2008a; Wang et al. 2010; Zhu et al. 2015). Furthermore, miRNA-171 has been discovered to control the reaction to different non-living environmental factors like salinity, drought, and heat in plants (Sunkar and Zhu 2004; Nguyen et al. 2015; Vakilian 2020).

Visentin et al. (2020) revealed a molecular link between drought and miR156 in tomato. In a previous study, drought recovery has been associated with controlling of stomatal behavior by miR156. Furthermore, miR156 has been found to regulate drought tolerance in alfalfa (Feyissa et al. 2019).

Mohsenifard et al. (2017) showed that the regulation of mir156 and mir396 under drought can induce drought tolerance by triggering GRF and MYB. In another study on rice, Nishad et al. (2022) determined that miRNA530 may play a vital role in salinity tolerance by changing its expression level.

### Expression levels of *PvMADS* genes in different tissues of *P. vulgaris*

**Fig. 9:**
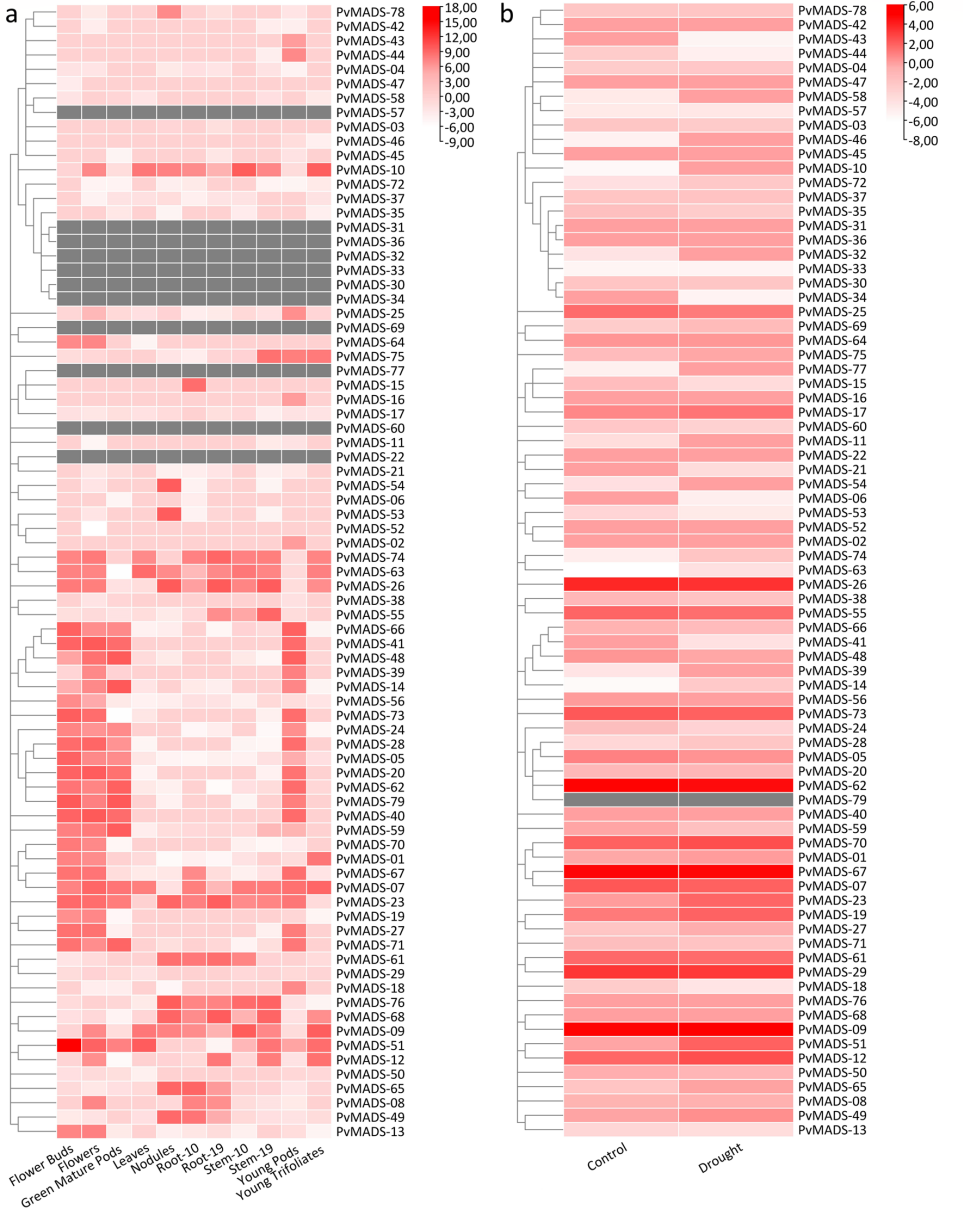
**a** Heatmap representing expression profiles of *PvMADS* in various tissues. **b** Heatmap representing the expression profiles of *PvMADS* under drought. The color scale shows log2FC values from high (red color) to low (white color). Twelve RNA samples were extracted in two replicates under either drought or control conditions. Gray rectangles indicate no data results

To examine the tissue-specific expression profiles of *PvMADS*, expression data in 11 tissues at different developmental stages were examined (Fig. 9a). According to the findings, expression levels were observed on different tissues with different levels Similarly, Zhao et al. (2021) demonstrated that the *SiMADS-box* genes had remarkably variable expression profiles in different plant tissues. According to the results of our study, among all *PvMADS* genes, *PvMADS-51* showed the highest expression level in flower buds. Intron-rich *PvMADS-07* is the gene with relatively higher expression levels in tissues than the other *PvMADS* genes. The genes that exhibit high expression levels in most of the plant tissues were generally found to contain larger introns. For example, *PvMADS-74* contains the longest intron, which exhibits high expression level in different tissues. It was also suggested by Zhou et al. (2023) that the genes which have more or longer introns are expressed more than others in plant cells.

### Investigation of *PvMADS* gene expression levels against drought stress by RNAseq data and RT-qPCR analysis

Transcription factors and hormones regulate important aspects of growth, development, and environmental stress responses. Plant development and survival are significantly impacted by abiotic stressors such as salinity, heat, cold, and flooding. To overcome these situations, plants have developed adaptation, tolerance, complex sensing, and signaling mechanisms. To create strategies to shield plants from the detrimental impacts of abiotic stresses and to ensure future demand for herbal products, it is necessary to explore stress response mechanisms at molecular level (Nair et al. 2022; Waadt et al. 2022). Drought is a serious environmental constraint for plant productivity. Considering the climate crisis, its effects reach a devastating level (Farooq et al. 2018). Reactive oxygen species (ROS) production is inevitably increased under drought stress in various cellular compartments, specifically in chloroplasts and mitochondria. Nonetheless, when faced with drought, plants elicit an extensive array of reactions, spanning from physiological, biochemical, and molecular levels (Kaur and Asthir 2017). In the light of this information, a heat-map graph was created using drought RNAseq data as described in M&M section (Fig. 9b). Accordingly, expression changes in *PvMADS* genes were investigated between control and drought stress conditions. As a result of the analysis, *PvMADS-10*, *PvMADS-32*, *PvMADS-46*, *PvMADS-58*, *PvMADS-77* genes were found to be upregulated dramatically in response to drought (Fig. 9b).

**Fig. 10:**
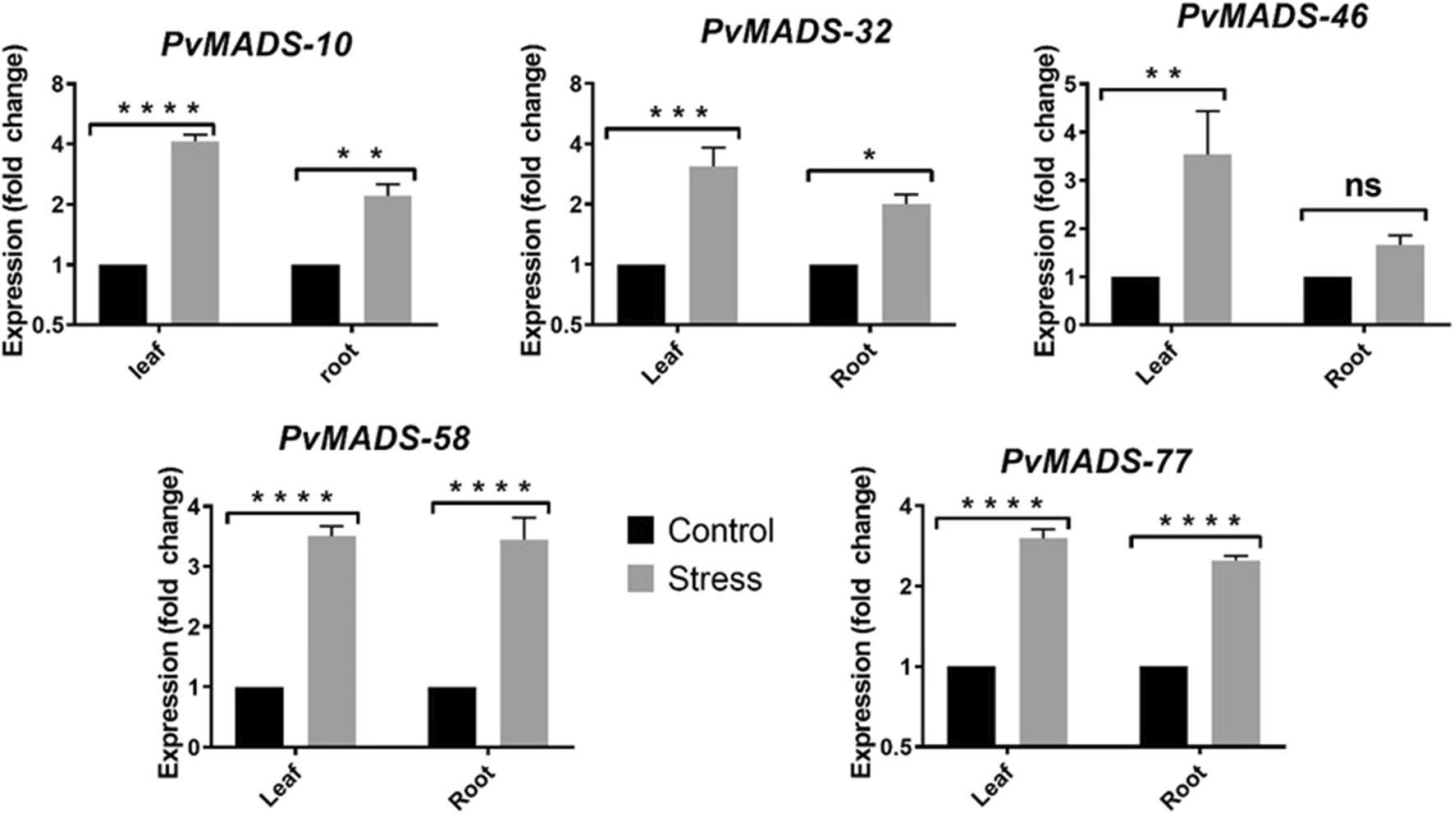
RT-qPCR analysis of selected candidate *PvMADS* genes. Relative gene expression levels of PvMADS genes in leaf (shown as black box) and root (shown as gray box) tissues. Errors bars represent the standard errors of three biological and two technical replicates, and asterisks signify the level of statistical significance (*p < 0.05, **p < 0.01, ***p < 0.001, ****p < 0.0001, ns = non-significant)

According to studies in different plant species, abiotic stress response and hormone regulation mechanisms are regulated by *MADS-box* genes (Castelán-Muñoz et al. 2019; Khong et al. 2015). To deeply understand the roles of *PvMADS* genes in drought response, their expression profiles were assessed using RT-qPCR technique, and 5 *PvMADS* genes (type I *PvMADS* genes: *PvMADS-10*, *PvMADS-32*, *PvMADS-46*, *PvMADS-58*, *PvMADS-77*) were found to be upregulated in both leaf and root tissues in consistent with RNAseq data (Fig. 10).

In a previous study, Zhao et al. (2021) were generated transgenic Arabidopsis and rice lines by overexpressing *SiMADS51* gene, and they were then subjected those lines to drought stress. As a result of their study, the overexpression of *SiMADS51* has been shown to led to drought sensitivity in *Arabidopsis* and rice lines. Saha et al. (2015) found that 8 *BrMADS* genes were induced in *B. rapa* against drought stress (Abbas et al. 2014). Additionaly, Yin et al. (2017) measured the physiological parameters of WT and transgenic plants in order to better evaluate the damage caused by drought stress, and it was revealed that *MADS-box* genes increased drought tolerance in tomato. Similar to those previous findings in the literature, we suggest that the *PvMADS* genes may have important roles in abiotic stress response in *P. vulgaris*. In addition, the study by Ayra et al. (2021) revealed that *MADS-box* genes are positive regulators of the bean-rhizobia symbiosis with functions such as root development, rhizobial infection, nodule organogenesis/function, and autoregulation of nodulation.

## Conclusions

This study offers insight into the *MADS* genes in the common bean. A total of 79 *PvMADS* genes were identified. Exon–intron and motif structure and phylogenetic relationships with *A. thaliana* were characterized. Considering evolutionary relationships, *PvMADS* genes were divided into two main classes as type I and type II. The MADS domain was found in type I and type II PvMADS proteins, while the K domain was found to be specific to type II PvMADS proteins. All chromosomes of the common bean were discovered to contain *PvMADS* genes. The orthologs of *A. thaliana*, *G. max*, and *V. unguiculata* provide an evolutionary insight, indicating that expression profiles in different tissues may be involved in developmental stages. Information from RNA-seq data was confirmed by RT-qPCR, indicating their potential role in stress regulation, revealing their involvement in drought stress tolerance. Overall, the data gave a clearer picture of the *PvMADS* genes in common bean, which could then be used to illustrate how they affect growth and development and increase the level of resistance to drought conditions. This study might be a reference for the future studies with *MADS* genes in different plant species.

### Supplementary Information

Below is the link to the electronic supplementary material.

Supplementary file1 (XLSX 222 KB)

Supplementary file2 (XLSX 12 KB)

Supplementary file3 (XLSX 21 KB)

Supplementary file4 (XLSX 373 KB)

Supplementary file5 (XLSX 13 KB)

Supplementary file6 (XLSX 9 KB)

## Data Availability

All data generated or analyzed during this study are included in this published article.
